# Method and Verification of Liquid Cooling Heat Dissipation Based on Internal Heat Source of Airborne Long-Focus Aerial Camera

**DOI:** 10.3390/s24206714

**Published:** 2024-10-18

**Authors:** Ziming Yuwen, Xinyang Li, Guoqin Yuan, Haixing Li, Jichao Zhang, Mingqiang Zhang, Yalin Ding

**Affiliations:** 1 Changchun Institute of Optics, Fine Mechanics and Physics, Chinese Academy of Sciences, Changchun 130033, China; 2 University of Chinese Academy of Sciences, Beijing 100049, China; 3 State Key Laboratory of Dynamic Optical Imaging and Measurement, Changchun 130033, China

**Keywords:** temperature gradient, aerial camera, liquid cooling heat dissipation, thermal analysis

## Abstract

The traditional passive heat dissipation method has low heat dissipation efficiency, which is not suitable for the heat dissipation of the concentrated heat source inside the long-focal aerial camera, resulting in temperature level changes and temperature gradients in the optical system near the heat source, which seriously affect the imaging performance of the aerial camera. To solve this problem, an active heat dissipation method of liquid cooling cycle is proposed in this paper. To improve the solving efficiency and ensure simulation accuracy, a dynamic boundary information transfer method based on grid area weighting is proposed. The thermal simulation results show that the liquid cooling method reduces the heat source temperature by 70.12%, and the boundary temperature transfer error is 0.015%. The accuracy of thermal simulation is verified by thermal test, and the simulation error is less than 6.44%. In addition, the performance of the optical system is further analyzed, and the results show that the MTF of the optical system is increased from 0.077 to 0.194 under the proposed active liquid cooling cycle heat dissipation method.

## 1. Introduction

Aerial camera is an important photoelectric payload for acquiring ground information and is widely used in UAS photogrammetry, resource exploration, target location and recognition, and other fields [1,2,3,4,5,6,7]. During the process of the airborne aerial camera reaching the working height from the ground, it experiences complex and violent external environmental disturbance, which directly affects the imaging performance of the optical system [8,9]. Ambient temperature disturbance changes the temperature level of the optical system, and the lens produces temperature gradient, which will aggravate the aberration and is a key factor affecting the optical performance [10,11,12,13]. Therefore, to ensure high-quality imaging from aerial cameras, it is important to use a temperature control method that maintains the optical system’s temperature level and reduces temperature gradients based on environmental conditions and internal structural constraints of the aerial camera.

The temperature control methods of aerial cameras under the influence of external ambient temperature have been extensively studied. Cheng [14] studied an active–passive composite temperature control technology, which maintained the temperature level of the primary mirror, and the temperature gradient between the primary mirror and the secondary mirror was less than 5 °C. Liu [15] proposed an active temperature control method to improve the temperature uniformity of the optical system, which improved the imaging resolution from 47 lp/mm to 59 lp/mm. Liu [16] carried out the thermal design of the main optical system of the aerial camera, so that the maximum temperature difference was less than 5 °C, and the thermal test results verified the accuracy of the thermal simulation. Yang [17] proposed an environmental stability design method based on a multi-dimensional structure, which makes the radial temperature difference of the optical system less than 0.5 °C and reduces the influence of pressure change at the same time. Liu [18] improved the temperature control scheme of the aerial camera such that the temperature uniformity was increased from 12.8 °C to 4.5 °C and clear images could be obtained at low pressure (35.4 kPa).

These studies address the issue of aerial camera optical performance degradation due to external temperature but overlook the impact of internal heat sources on the optical system. There are few studies on the heat dissipation of concentrated heat sources inside long-focal aerial cameras. Gao [19] used thermoelectric refrigeration devices to control the temperature of the lenses in the aerial camera, while using phase change components to dissipate heat from the heat source, keeping the optical system at a stable temperature level. Li [20] studied the influence of active and passive combined temperature control measures on the temperature level of the optical system and adopted phase change heat dissipation for the CCD components inside the aerial camera—after 2 h, the temperature rise in the CCD components decreased from 23.4 °C to 10 °C, ensuring the imaging quality. However, the cooling efficiency of the thermoelectric refrigeration structure is low, which is only suitable for the heat dissipation of low-power devices, and the arrangement is limited by the structure of the aerial camera. Phase change components do not effectively dissipate heat for high-power devices over the long term. They are unable to transfer heat outside of the camera, which can lead to unstable temperatures inside the camera. As aerial cameras advance toward longer focal lengths and higher resolutions, the optical system becomes more sensitive to heat. Traditional passive heat dissipation methods are no longer appropriate.

The liquid cooling method has the advantages of high heat dissipation efficiency and good controllability and is widely used in the thermal management of edge data center servers, new energy vehicle fuel cell stacks, motor cooling for unmanned aerial vehicles, 3-D printable antenna array, and other fields [21,22,23,24,25,26]. So far, there are no detailed simulations or experimental results on liquid cooling methods for aerial cameras in the literature. To maintain the temperature stability of the aerial camera’s optical system in this paper, we propose a liquid cooling system suitable for the internal concentrated heat source of long-focus aerial cameras, develop a method for dynamic transmission of boundary information for liquid cooling circulation simulation, and produce a prototype liquid cooling test piece.

The subsequent arrangement of this paper is as follows: In the second section, the layout of the liquid cooling structure for the aerial camera and heat transfer theoretical model adopted are introduced, and a boundary information dynamic transfer method based on grid area weighting is proposed. In the third section, the thermal simulation model is established and the thermal simulation and thermal test are carried out. In the fourth section, the optical performance is analyzed. The fifth section is the conclusion.

## 2. Models and Methods

### 2.1. Liquid Cooling Structure Layout of the Aerial Camera

**Figure 1:**
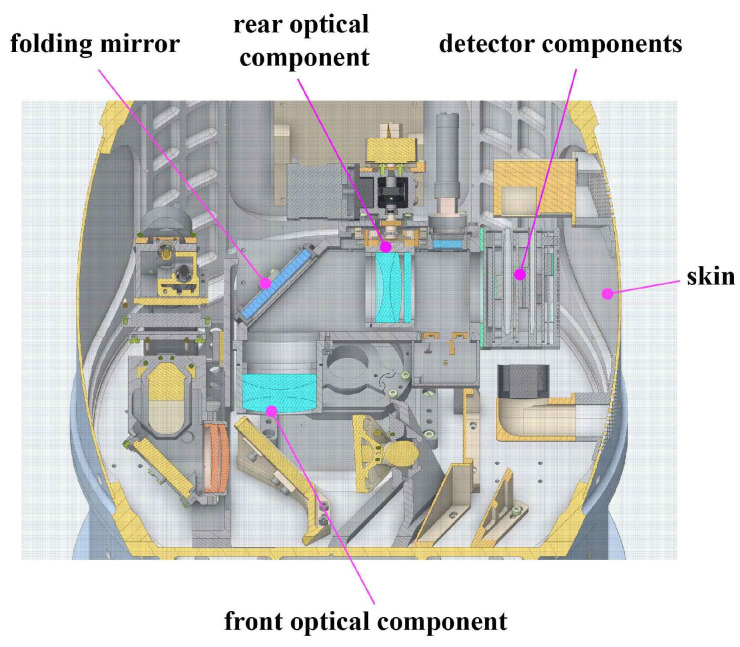
Diagram of the middle assembly of the aerial camera.

The working altitude of the airborne aerial camera is 8 km, with extreme temperature and pressure conditions of 0 °C and 36 kPa. The optical system consists of a main optical system and a visible light optical subsystem, with a focal length of up to 2 m. The detector components, as a concentrated heat source, are located near the optical subsystem in the middle assembly of the aerial camera, with a thermal power consumption of 16 W. The internal model of the middle assembly of the aerial camera is shown in Figure 1. The middle assembly is covered with four skins, and the optical subsystem is composed of front optical components, a folding mirror, and rear optical components. Under the continuous operating conditions of the aerial camera, the temperature level of the optical subsystem changes, resulting in uneven thermal stress on the lens surface, which affects the imaging performance of the optical system.

**Figure 2:**
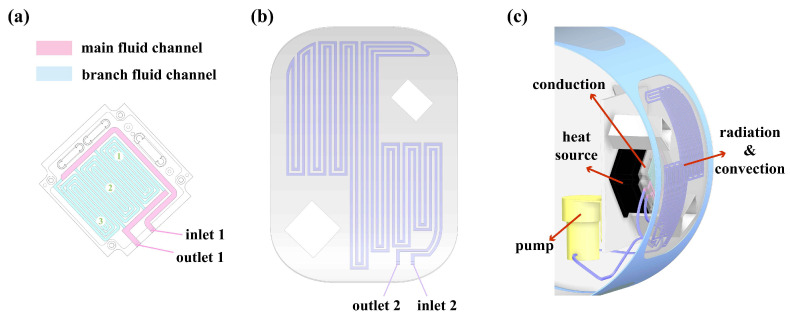
Liquid cooling structure: (**a**) heat absorber; (**b**) heat-dissipation skin; (**c**) schematic diagram of heat dissipation.

The aerial camera has strict volume and weight constraints. Based on the aerial camera’s structural characteristics, the heat source’s back cover is selected as the heat absorber, and the skin closest to the heat source is used as the heat-dissipation skin. Figure 2a shows the fluid channel inside the heat absorber, with a cross-sectional size of 4 mm × 1.5 mm for the main fluid channel. The coolant flows through branch fluid channels 1, 2, and 3 in the direction of the mainstream, respectively. The cross-sectional size of the branch fluid channel is 2 mm × 1.5 mm. Figure 2b shows the heat-dissipation skin with a curvature radius of 210 mm and a thickness of 2 mm. To fully utilize the heat dissipation area of the skin, the fluid channel adopts a tightly folded structure with a cross-sectional size of 3 mm × 1 mm, and the outlet and inlet sizes are both 4 mm × 3 mm. For the convenience of subsequent liquid cooling heat dissipation test verification, the middle assembly of the aerial camera is simplified, and only the heat source and heat dissipation structure are retained inside. As shown in Figure 2c, the inlet and outlet of each device are connected by hoses, and the inlet velocity of the coolant is controlled by a pump. The heat absorber absorbs heat through thermal conduction, and the coolant temperature rises and is transferred to the heat-dissipation skin, which dissipates heat externally by radiation and convection. The heat absorber and the heat-dissipation skin are made of aluminum alloy, the frame material of the middle assembly is ZTC4, and the coolant is n-octane. The thermophysical properties of materials are shown in Table 1. 

### 2.2. Theoretical Model

#### 2.2.1. Heat Transfer Model

In the heat dissipation process of the concentrated heat source inside the aerial camera, the liquid-cooled forced convection heat dissipation is dominant. However, contact thermal resistance in heat conduction, natural convection in the air domain inside the middle assembly, and thermal radiation from the heat source also affect the accuracy of the thermal simulation, where contact thermal resistance can be expressed as

(1) $$R=\frac{1}{K\cdot S}$$

where K (W/m^2^·K) is the contact thermal resistance coefficient and S (m^2^) is the contact area.

The air near the heat source is affected by temperature differences, causing density changes, so the incompressible Navier–Stokes equation is no longer applicable. Since the temperature rise in the internal electronic devices in aerial cameras usually does not exceed 100 °C, the change in air density is small, the natural convection meets the Boussinesq hypothesis, and then the N–S equation is as follows:

(2) $$\frac{\partial U}{\partial t}+\nabla \cdot \left(UU\right)=-\nabla p_{k}+v\nabla^{2}U+\left(1-\beta \left(T-T_{0}\right)\right)g$$

In Equation (2),

(3) $$p_{k}=\frac{p}{\rho_{0}},\;\beta =-\frac{1}{\rho_{0}}{\left(\frac{\partial \rho }{\partial T}\right)}_{p}\approx -\frac{1}{\rho_{0}}\frac{\rho -\rho_{0}}{T-T_{0}}$$

where $p_{k}$ is the kinematic pressure, $\rho_{0}$ is the reference density, and β is the coefficient of thermal expansion.

The thermal radiation of the heat source and surrounding components is calculated using the Surface-to-Surface (S2S) thermal radiation model, which obtains the actual temperature distribution by calculating the view factor between surfaces. The model can be expressed as

(4) $$\left[\begin{array}{ccccc} 1 & \left(\varepsilon_{1}-1\right)F_{12} & \left(\varepsilon_{1}-1\right)F_{13} & \cdot \cdot \cdot & \left(\varepsilon_{1}-1\right)F_{1N}\\ \left(\varepsilon_{2}-1\right)F_{21} & 1 & \left(\varepsilon_{2}-1\right)F_{23} & \cdot \cdot \cdot & \left(\varepsilon_{2}-1\right)F_{2N}\\ \left(\varepsilon_{3}-1\right)F_{31} & \left(\varepsilon_{3}-1\right)F_{32} & 1 & \cdot \cdot \cdot & \left(\varepsilon_{3}-1\right)F_{3N}\\ \cdot \cdot \cdot & \cdot \cdot \cdot & \cdot \cdot \cdot & \cdot \cdot \cdot & \cdot \cdot \cdot \\ \left(\varepsilon_{N}-1\right)F_{N1} & \left(\varepsilon_{N}-1\right)F_{N2} & \left(\varepsilon_{N}-1\right)F_{N3} & \cdot \cdot \cdot & 1 \end{array}\right]\left(\begin{array}{c} q_{1,out}\\ q_{2,out}\\ q_{3,out}\\ \cdot \cdot \cdot \\ q_{N,out} \end{array}\right)=\left(\begin{array}{c} \varepsilon_{1}\sigma T_{1}^{4}\\ \varepsilon_{2}\sigma T_{2}^{4}\\ \varepsilon_{3}\sigma T_{3}^{4}\\ \cdot \cdot \cdot \\ \varepsilon_{N}\sigma T_{N}^{4} \end{array}\right)$$

where N is the number of grid surfaces, $\varepsilon_{k}$ is the emissivity of the surface k(k=1,2,⋅⋅⋅,N), $q_{k,out}$ is the energy flux leaving the surface k, σ is the Stefan–Boltzmann constant, and $T_{k}$ is the temperature of the surface k. The view factor $F_{jk}$ is the fraction of energy leaving surface j that is incident on surface k given by the following formula:

(5) $$F_{jk}=\frac{1}{A_{j}}\int_{A_{j}}\int_{A_{k}}\frac{\cos\theta_{j}\cos\theta_{k}}{\pi r^{2}}\delta_{jk}dA_{j}dA_{k}$$

where $A_{j}$ and $A_{k}$ are the area of surface j and surface k; r is the length connecting $A_{j}$ and $A_{k}$; $\theta_{j}$ and $\theta_{k}$ are the angles formed by r with surface j and surface k, respectively; $\delta_{jk}$ is determined by the visibility of $dA_{k}$ to $dA_{j}$. If $dA_{k}$ is visible to $dA_{j}$, then $\delta_{jk}$ = 1; otherwise, $\delta_{jk}$= 0.

#### 2.2.2. Turbulence Model

The turbulence model can be divided into zero equation model, single equation model, and two-equation model according to the number of transport equations. The zero-equation model is characterized by simple theory and fast solving speed, but low solving accuracy for complex flow fields. The single equation model is suitable for solving the flow with inverse pressure gradient near the wall; since only the transport equation for turbulent kinetic energy k needs to be solved, the solving speed is relatively fast. At present, the standard k−ε model is widely used in the engineering field—that is, the turbulent dissipation rate ε transport equation is introduced on the basis of the turbulent kinetic energy k transport equation. Although the standard k−ε two-equation model has good performance in many fields, it is not very accurate for swirl flow, reflux, and boundary layer separation. The RNG k−ε model is derived from the renormalization group theory and is similar in form to the standard k−ε model. The difference is that the RNG k−ε model revises the turbulent viscosity $\mu_{t}$ and turbulent dissipation rate ε, takes into account the effect of low Reynolds number, and improves the accuracy of swirling flow and high-speed flow. In this paper, the RNG k−ε model is selected to simulate the flow field. The transport equations of turbulent kinetic energy k and turbulent dissipation rate ε are as follows:

(6) $$\frac{\partial \left(\rho k\right)}{\partial t}+\frac{\partial \left(\rho ku_{i}\right)}{\partial x_{i}}=\frac{\partial }{\partial x_{j}}\left(\alpha_{k}\mu_{eff}\frac{\partial k}{\partial x_{j}}\right)+G_{k}-\rho \varepsilon$$

(7) $$\frac{\partial \left(\rho \varepsilon \right)}{\partial t}+\frac{\partial \left(\rho \varepsilon u_{i}\right)}{\partial x_{i}}=\frac{\partial }{\partial x_{j}}\left(\alpha_{\varepsilon }\mu_{eff}\frac{\partial \varepsilon }{\partial x_{j}}\right)+\frac{C_{1\varepsilon }\varepsilon }{k}G_{k}-C_{2\varepsilon }\rho \frac{\varepsilon^{2}}{k}-R_{\varepsilon }$$

where $G_{k}$ is the generation term of turbulence kinetic energy due to the mean velocity gradients and $R_{\varepsilon }$ is the additional term in equation ε. The quantities $\alpha_{k}$ and $\alpha_{\varepsilon }$ are the inverse effective Prandtl numbers for k and ε, respectively. $\mu_{eff}$ is the effective viscosity. $\mu_{eff}=\mu +\mu_{t}$ and $\mu_{t}=\rho C_{\mu }\frac{k^{2}}{\varepsilon }$. Model constants are as follows:

$$C_{\mu }=0.0845,\;\alpha_{k}=\alpha_{\varepsilon }=1.39;\;C_{1\varepsilon }=1.42;\;C_{2\varepsilon }=1.68.$$

### 2.3. Dynamic Boundary Information Transfer Method Based on Grid Area Weighting

The internal structure of the pump is complex, and if the precise geometric details inside are considered when establishing a thermal analysis model, it will lead to a serious decrease in computational efficiency and even solution divergence. If the pump model is not considered and simulation is conducted directly, the temperature information in the fluid domain cannot be connected and the temperature information at the outlet boundary cannot be updated to the inlet promptly, resulting in simulation results deviating from the actual situation. To solve this problem, an equivalent treatment is applied to the connectivity of the pump during the simulation process, which omits the pump and directly connects inlet 1 and outlet 2 through a program.

**Figure 3:**
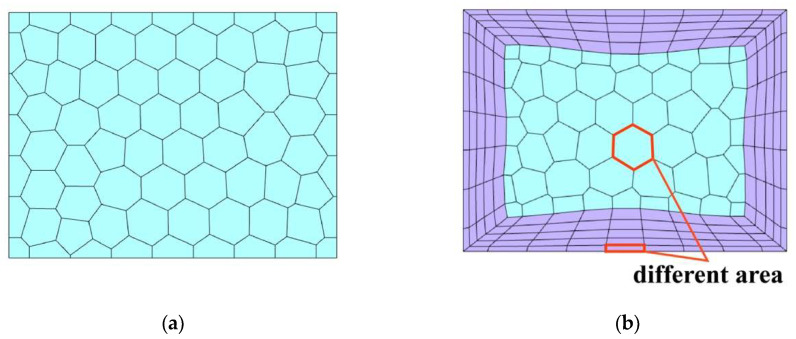
Outlet 2 grid distribution forms: (**a**) without boundary layer; (**b**) boundary layer refinement.

The grid distribution forms of outlet 2 are exhibited in Figure 3. Figure 3a is a grid division form without boundary layer, in which the boundary temperature is obtained by taking the arithmetic mean of the temperature information stored on all grid surfaces. Figure 3b shows the grid form of fluid boundary layer refinement. Due to the high temperature gradient of the boundary layer, the temperature information stored on each grid surface in the boundary layer varies greatly along the direction perpendicular to the wall. In addition, the area of the boundary layer grid is usually much smaller than that of the mainstream zone. Therefore, only calculating the arithmetic mean of the boundary temperature will result in significant errors. To ensure the accuracy of the transmission of boundary temperature information, an area-weighted form is adopted to calculate the outlet 2 boundary temperature information.

**Figure 4:**
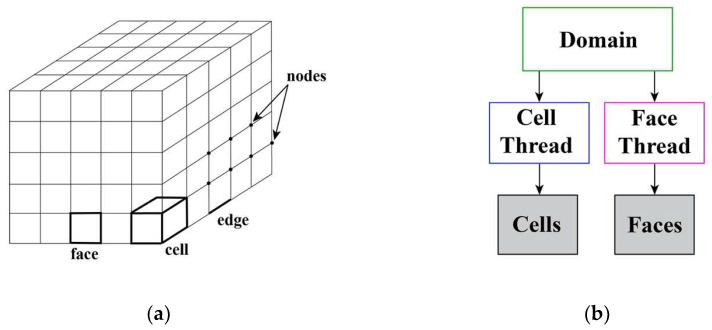
Grid components and data structure: (**a**) compute domain 3D grid; (**b**) the relationship between data structures and threads.

In the simulation, the grid model is divided into cells, as illustrated in Figure 4a. Each cell is defined by a set of nodes, a cell center, and the faces that bind the cell. The cells and cell faces are grouped into different zones that are used to define fluid inlets, outlets, walls, and fluid compute domain information. Figure 4b shows the relationship between data structures and threads. The domain is a data structure used to store information about the collection of nodes, face threads, and cell threads in the grid. Face threads and cell threads are data structures used to store information about boundaries and cell zones, respectively. Moreover, each boundary or cell zone defined for the thermal analysis model has an integer ID corresponding to it, and information about the boundary or cell zone represented by each thread can be accessed through pointers pointing to the thread.

**Figure 5:**
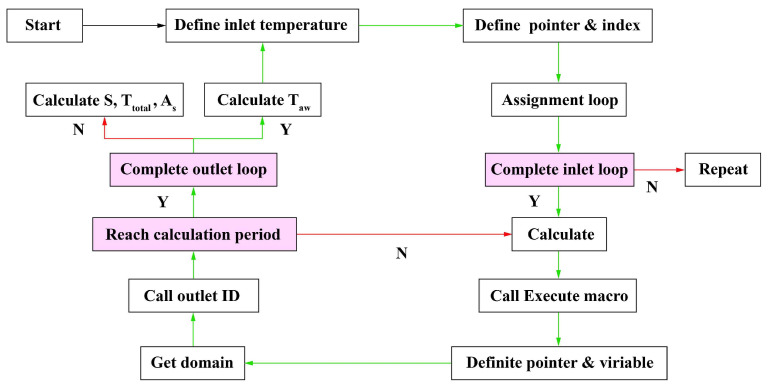
Flowchart of dynamic boundary information transfer method based on grid area weighting.

As shown in Figure 5, the method is based on the cells and thread pointers of the fluid computing domain and can access the cell data and face data of the computing domain by calling predefined macros. The influence of the stored information of each grid face on the temperature boundary is considered, and dynamic adjustment is made through iterative convergence judgment. The process is as follows:

1. Define the initial temperature $T_{in}$ = 273.15 (K) at the inlet boundary.
2. Define the pointer t to the inlet boundary face thread and the temperature variable index i, which are passed by the solver to the program.
3. Define the grid face variable f, loop each face f in the inlet face thread t, index all grid faces in the loop, and complete the initial temperature boundary assignment.
4. Call the execution macro after each iteration calculation, define the fluid domain variable d and the face variable f, and call the thread corresponding to the fluid domain outlet boundary face ID. The update period of the inlet boundary is defined according to the number of steps that can reach convergence. Define variables $T_{total}$ (representing the sum of the product of each grid face area and its corresponding stored temperature information), $T_{aw}$ (representing the average temperature value weighted based on the grid faces area at the fluid outlet boundary), $A_{s}$ (representing the sum of grid faces area at the fluid outlet boundary), and define array A to store the area data.
5. When the fluid domain pointer is not explicitly passed to the program as a parameter, the fluid domain of the calculation domain is obtained by retrieving the domain pointer; the thread pointer points to the fluid domain outlet boundary face zone ID to avoid the fluid outlet boundary missing due to the randomness of each thread during parallel computation.
6. When the number of iterations meets the convergence period, the face loop on the fluid outlet boundary face thread is executed. The actual area of a given face f in the face thread t can be calculated by Equation (8), where x[0], x[1], and x[2] are the three components of the area vector. Multiply and sum the area vector with the corresponding face temperature to obtain Equation (9), where n is the number of face elements on the outlet boundary and $S_{k}$ is the area of the face k. $T_{k}$ is the temperature information stored by the face k, and Formula (10) represents the sum of the area on the outlet boundary face region.
  (8) $$S=\sqrt{x\left[0\right]*x\left[0\right]+x\left[1\right]*x\left[1\right]+x\left[2\right]*x\left[2\right]}$$
  (9) $$T_{total}=\sum_{k=1}^{n}S_{k}\cdot T_{k}$$
  (10) $$A_{s}=\sum_{k=1}^{n}S_{k}$$
7. The average temperature value at the fluid outlet boundary weighted by grid area can be expressed as
  (11) $$T_{aw}=\frac{T_{total}}{A_{s}}$$

The outlet temperature information obtained by Equation (11) can be assigned to the inlet to complete a liquid cooling cycle, and the program can be dynamically adjusted according to the outlet temperature changes in the iterative calculation process.

## 3. Thermal Simulation and Thermal Test

### 3.1. Thermal Simulation Analysis

**Figure 6:**
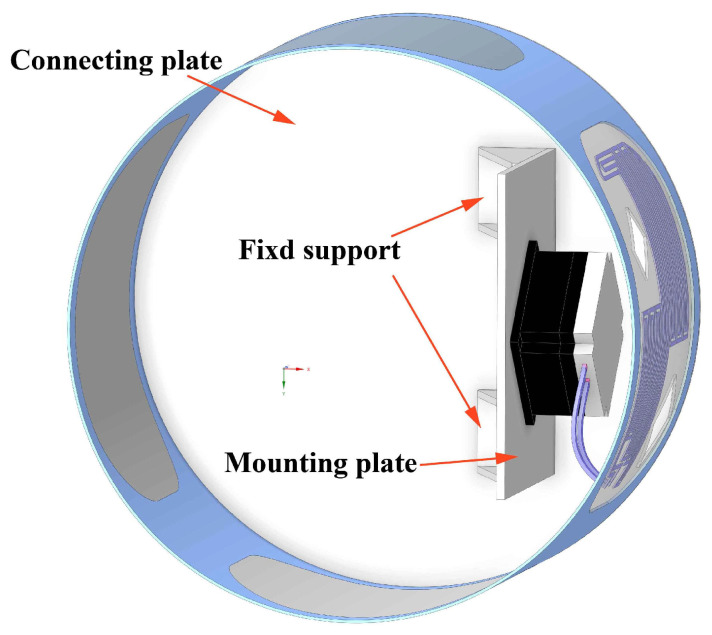
Simplified model of the middle assembly of the aerial camera.

The simplified model of the middle assembly after equivalent treatment of the pump is shown in Figure 6. The heat source is fixed on the mounting plate, and the mounting plate is connected to the connecting plate through the support structure.

**Figure 7:**
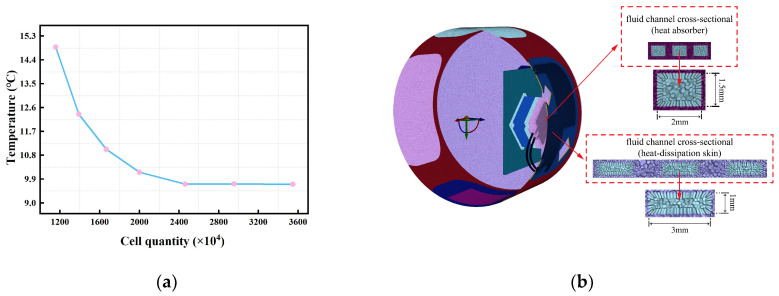
Grid division: (**a**) grid independence verification; (**b**) grid model for thermal analysis.

The grid density of the liquid cooling fluid channel determines whether the precise information of the physical field gradient can be captured during the calculation process. To ensure the simulation’s accuracy, grid independence verification was performed. Figure 7a shows the grid independence verification results. When the cell quantity is 11.57 million, the heat source temperature is 14.88 °C at thermal equilibrium. When the cell quantity increased, the heat source temperature changed. When the cell quantity reached 24.64 million, the temperature of the detector was 9.71 °C; then, the cell quantity continued to increase and the simulation results were basically unchanged. Therefore, in order to save computing resources, the cell quantity was 24.64 million and the grid orthogonality was higher than 0.23. The grid model for thermal analysis is shown in Figure 7b.

The inlet boundary is set as velocity inlet. The inlet temperature changes dynamically with the outlet temperature and is automatically regulated by the program. The outlet boundary uses a pressure outlet, and the parameters remain default. The heat generation rate of the heat source is 70,083 W/m^3^, and the natural convection and thermal radiation are calculated using the model in Section 2.2.1. The SIMPLE algorithm is used for the pressure–velocity coupling. The momentum and energy equations are discretized by the second-order upwind scheme, and the governing equations of turbulent kinetic energy and turbulent dissipation rate are discretized by the second-order upwind scheme as well. The initial temperature is 273.15 K, and the operating pressure is 36 kPa.

**Figure 8:**
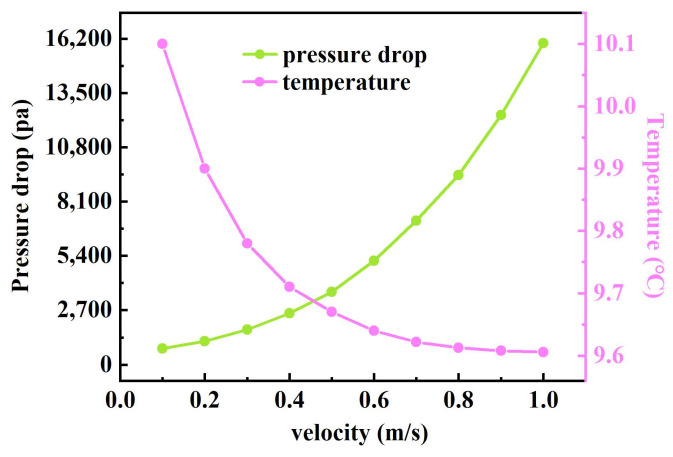
Pressure drop and temperature change with inlet velocity.

The pressure drop and heat source temperature change under different inlet velocities are simulated. As can be seen from Figure 8, with the increase in the coolant’s inlet velocity, the pressure drop increases and the increasing rate gradually becomes larger. Meanwhile, the heat source temperature shows a decreasing trend with the increase in the velocity and the decreasing rate gradually becomes smaller. To avoid excessive pump power consumption, we choose an inlet velocity of 0.4 m/s.

**Figure 9:**
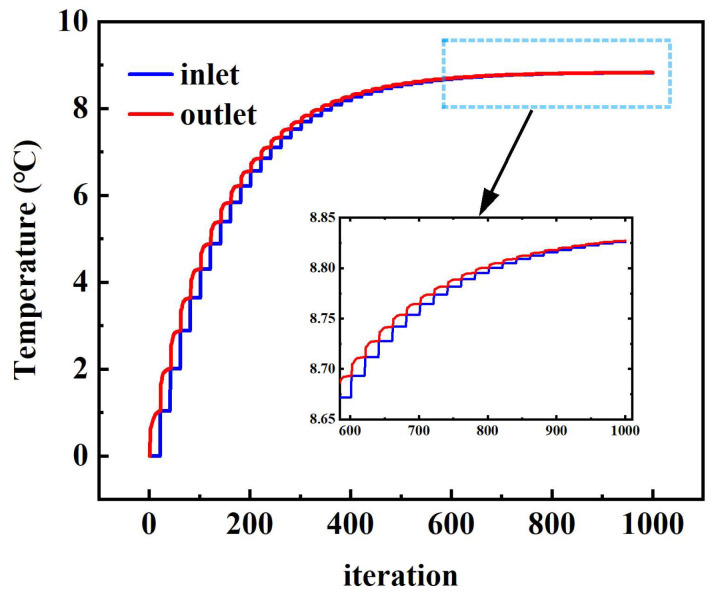
Temperature changes at the coolant inlet and outlet.

Figure 9 shows the temperature changes at the inlet and outlet at an inlet speed of 0.4 m/s. It can be seen that the temperature at the inlet boundary of the coolant is dynamically updated with the temperature at the outlet during each iteration period. After 400 iterations, the heat dissipation process gradually approaches the thermal equilibrium state. The temperature values and errors of the coolant inlet and outlet are shown in Table 2. After dynamic adjustment by the program, the error between the inlet boundary temperature and the outlet boundary temperature is 0.015%, and the error comes from the interpolation error between grid nodes caused by the non-shared topology of the inlet and outlet boundary faces. The simulation results verify the accuracy of the proposed dynamic transfer method of temperature boundary information based on area weighting.

**Figure 10:**
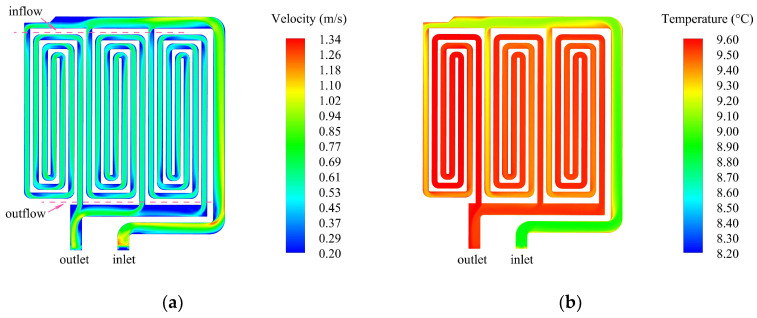
The cross-section of the heat absorber: (**a**) velocity distribution; (**b**) temperature distribution.

Figure 10a shows the velocity distribution of the fluid channel cross-section of the heat absorber. It can be seen from the figure that when the coolant enters from the inlet, the velocity increases to a maximum of 1.34 m/s—this occurs because the cross-section at the inlet is larger; then, the fluid channel quickly narrows due to the constant flow rate at the inlet and the velocity of the coolant passing through the narrow channel increases. When the coolant passes through each branch fluid channel, the velocity of the mainstream decreases to a certain extent due to the split flow. “Inflow” in the figure corresponds to the cross-section position of the mainstream flowing into the three branch fluid channels. “Outflow” is the cross-section position where the three branch fluid channels meet with the main fluid channel, respectively. The flow rates at the corresponding six cross-section locations are given in Table 3. It can be seen that the inflow and outflow of the three branch fluid channels are basically consistent. Moreover, it can be seen from the velocity contour diagram that the coolant at different branch fluid channels has the same velocity distribution, indicating that the heat absorber carries out uniform convection heat transfer. Figure 10b shows the temperature distribution of the fluid channel cross-section of the heat absorber. It can be seen that the inlet temperature of the coolant is 8.8 °C, and the temperature rises slowly with the extension of the distance of the fluid channel from the inlet. The coolant in the branch fluid channels has the same temperature distribution, and the outlet temperature is 9.6 °C, which is 0.8 °C higher than the inlet temperature.

**Figure 11:**
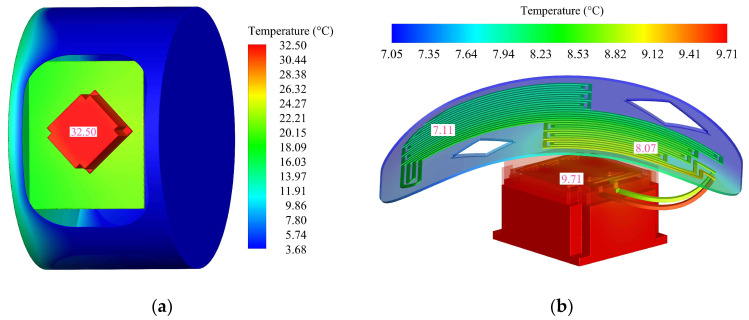
Temperature simulation results: (**a**) natural heat dissipation; (**b**) liquid cooling heat dissipation.

The temperature distribution after 2 h of natural heat dissipation is shown in Figure 11a, and the temperature of the heat source reaches 32.50 °C. Figure 11b shows the temperature distribution when liquid cooling is in thermal equilibrium. The temperature at the inlet of the heat-dissipation skin is 8.07 °C, and the temperature drops to 7.11 °C after the heat is dissipated to the outside space through the skin, resulting in a temperature difference of 0.96 °C. The thermal equilibrium temperature of the heat absorber is 9.71 °C, and the temperature of the heat source is reduced by 70.12% compared with the natural heat dissipation state, which verifies the effectiveness of the liquid cooling heat dissipation method.

### 3.2. Thermal Test

**Figure 12:**
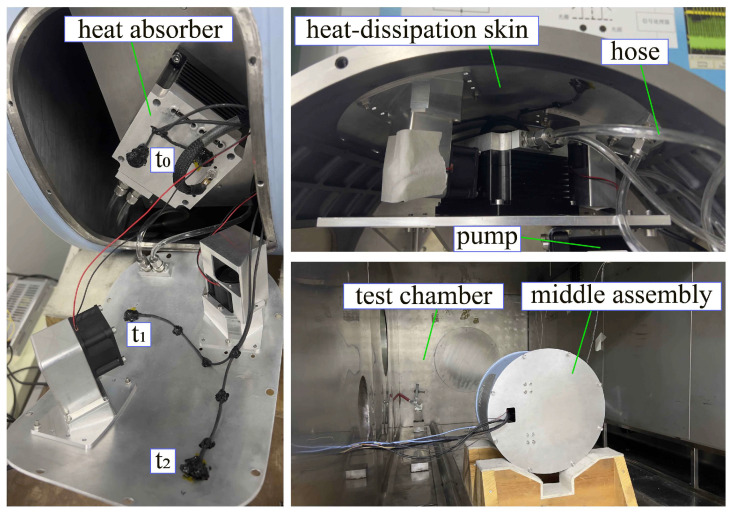
Schematic diagram of liquid cooling test for the middle assembly of the aerial camera.

To verify the accuracy of the thermal simulation in the previous section, the thermal test of the middle assembly of the aerial camera was carried out in low-temperature and low-pressure environments. As shown in Figure 12, temperature monitoring points t_0_, t_1_, and t_2_ are arranged on the heat absorber and heat-dissipating skin, respectively. The test piece was placed in the temperature–height test chamber to simulate the operating environment of 0 °C and 36 kPa. The test conditions were divided into natural heat dissipation and liquid cooling heat dissipation, and the opening and closing of liquid cooling heat dissipation measures were controlled by a computer program.

**Figure 13:**
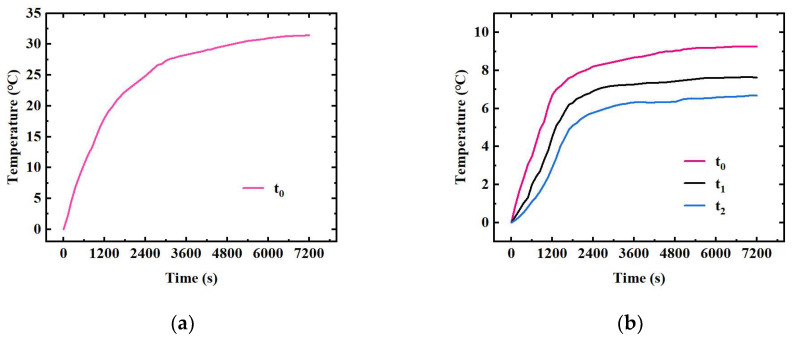
Temperature change under different operating conditions: (**a**) natural heat dissipation; (**b**) liquid cooling heat dissipation.

Figure 13a shows the temperature change under natural heat dissipation, and after 2 h of operation, the temperature of the heat source reached 31.45 °C. Figure 13b shows the temperature change under liquid cooling heat dissipation. After 2 h, the temperature of the heat source is 9.25 °C; the temperatures of the hot end and the cold end of the heat-dissipation skin are 7.63 °C and 6.68 °C, respectively; and the heat source temperature is reduced by 70.59% after liquid cooling heat dissipation. The comparison of temperature results between tests and simulations under different operating conditions is shown in Table 4, the simulation error is less than 6.44%, and the simulation results are in good agreement with the test results.

## 4. Optical System Simulation Analysis

**Figure 14:**
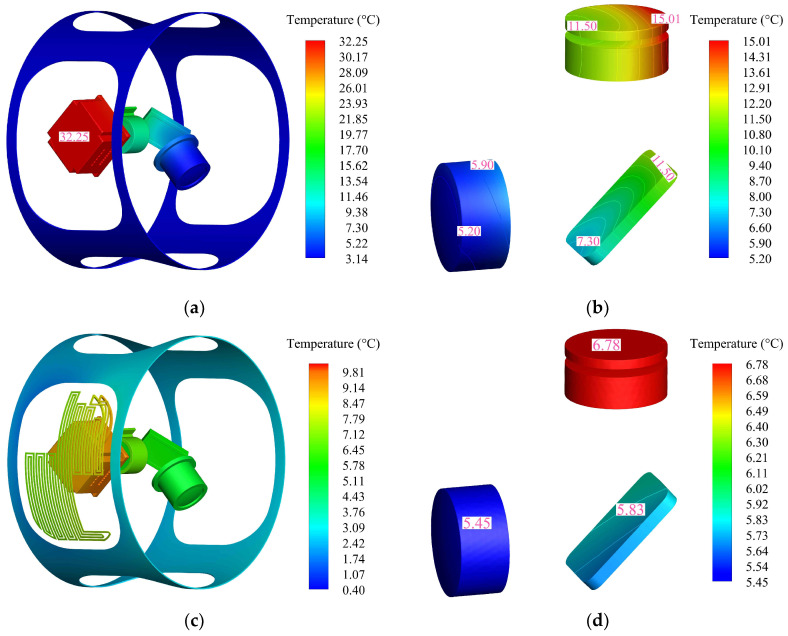
Temperature distribution: (**a**) heat source under natural heat dissipation; (**b**) optical subsystem under natural heat dissipation; (**c**) heat source under liquid cooling; (**d**) optical subsystem under liquid cooling.

The previous section verified the accuracy of the thermal simulation of the simplified model. Therefore, the optical performance of the actual middle assembly model of the aerial camera is analyzed in this section. Figure 14a,b show the temperature distribution of the heat source and optical subsystem in the middle assembly after operating for 2 h under natural heat dissipation. It can be seen that the temperature of the heat source at thermal equilibrium is 32.25 °C and the temperature level of the optical subsystem changes. There is also a temperature gradient in the lenses, with a maximum temperature difference of 9.81 °C. The temperature distribution under liquid cooling cycle heat dissipation conditions is shown in Figure 14c,d. It can be seen that the temperature level inside the middle assembly has significantly decreased, with the temperature of the heat source and the maximum temperature of the lens decreased by 69.58% and 54.83%, respectively. The temperature gradient of the lenses is effectively reduced, with a maximum temperature difference of only 1.33 °C, and the optical subsystem has good temperature uniformity.

**Figure 15:**
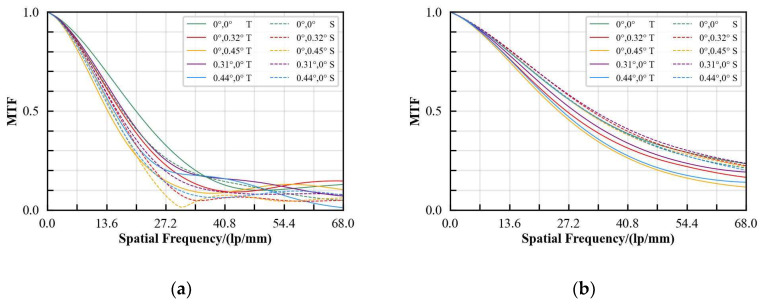
MTF of the optical system under different operating conditions: (**a**) natural heat dissipation; (**b**) liquid cooling heat dissipation.

The three-dimensional temperature field distribution of optical–mechanical components can be transferred to the mechanical analysis module through CFD interpolation; the rigid body motion and thermoelastic deformation of lens surface can be calculated through the thermal deformation data; and the optical properties under the influence of thermal structure can be analyzed by importing the data into Zemax in the form of a ZPL file [27,28]. The MTF of the optical system under different operating conditions is shown in Figure 15. The MTF at cut-off frequency under natural heat dissipation condition is 0.077, and the corresponding MTF under the liquid cooling heat dissipation condition is 0.194, which shows a significant improvement. This indicates that the liquid cooling cycle heat dissipation method can improve the optical imaging performance under the heat concentration inside the aerial camera.

## 5. Conclusions

The traditional passive heat dissipation method is no longer suitable for the long-focus, high-resolution aerial camera with volume and weight constraints. In this paper, a liquid cooling cycle heat dissipation method is proposed for the concentrated heat source inside the long-focus, high-resolution aerial camera. An area weighted boundary information transfer method is developed, which can realize the dynamic transfer of information during the simulation iteration process, and the boundary temperature transfer error is 0.015%. The thermal simulation results show that the proposed liquid cooling method can effectively reduce the temperature of the heat source, and the accuracy of the thermal simulation is verified by the thermal test. Furthermore, the integrated analysis method is used to analyze the optical performance of the optical system of the aerial camera. The analysis results show that the temperature gradient under the liquid cooling condition is effectively reduced, and the MTF is increased from 0.077 to 0.194.

It should be noted that the liquid cooling channel structure is designed according to the original structure of the aerial camera (detector shell and camera skin), and the connecting pipe is a hose that can be adjusted according to the specific structure, so these parts do not take up space. However, the size of the circulating pump may be limited by different camera models, resulting in additional space occupancy. The methods proposed in this paper can provide useful guidance for the active heat dissipation of the internal concentrated heat source in long-focus, high-resolution aerial cameras.

## Figures and Tables

**Table 1:** Thermophysical properties of materials.

Materials	Fusing Point	Boiling Point	Density	Specific Heat	Viscosity	Thermal Conductivity
	°C	°C	kg/m^3^	J/(kg·K)	Pa·s	W/(m·K)
Aluminum alloy	—	—	2800	904	—	142
ZTC4	—	—	4400	577	—	8.8
N-OCTANE	−57	125	762.39	2013.146	0.002166	0.153

**Table 2:** Temperature comparison of coolant inlet and outlet.

Inlet Temperature	Outlet Temperature	Error
8.82588 °C	8.82721 °C	0.015%

**Table 3:** The flow rate of different branch fluid channels.

Flow Rate (g/s)	Branch Channel 1	Branch Channel 2	Branch Channel 3
Inflow	1.220	1.216	1.224
Outflow	1.220	1.216	1.224

**Table 4:** Comparison of temperature results between tests and simulations.

Operating Conditions	Simulation (°C)			Test (°C)			Error		
natural heat dissipation	t_0_			t_0_			t_0_		
	32.50			31.45			3.34%		
liquid cooling heat dissipation	t_0_	t_1_	t_2_	t_0_	t_1_	t_2_	t_0_	t_1_	t_2_
	9.71	8.07	7.11	9.25	7.63	6.68	4.97%	5.77%	6.44%

## Data Availability

The data presented in this study are available on request from the corresponding author due to privacy.
